# A Case of Adenosarcoma of the Uterus

**DOI:** 10.1155/2014/342187

**Published:** 2014-01-28

**Authors:** Shigeki Taga, Mari Sawada, Aya Nagai, Dan Yamamoto, Ryoji Hayase

**Affiliations:** Department of Obstetrics and Gynecology, National Hospital Organization Fukuyama Medical Center, Okinogamicho 4-14-17, Fukuyama 720-0825, Japan

## Abstract

Adenosarcoma is a rare tumor which consists of benign glandular epithelium and malignant mesenchymal component. Here we report a case of adenosarcoma of the uterine corpus. *Case Presentation*. A 59-year-old woman presented with vaginal bleeding and visited a local clinic. She had a uterine tumor pointed out and was referred to our hospital. Ultrasound scans revealed a large heterogeneous mass occupying the whole uterine cavity. Cytological test of endometrium was performed but the result was negative. A fractional endometrial curettage revealed no malignancy. Magnetic resonance imaging (MRI) revealed a heterogeneous solid tumor of 77 × 76 mm. Total abdominal hysterectomy with bilateral salpingo-oophorectomy and pelvic lymphadenectomy was performed. On gross examination, the tumor was arising from the uterine body and occupied the whole uterine cavity. Histopathological examination revealed phyllodes-like architecture on low magnificationandperiglandular cuffing of tumor cells. The lesion was confined to the uterus. Histopathological final diagnosis was adenosarcoma. Her postoperative course was uneventful and she was discharged without postoperative treatment and remains alive without disease 6 months after the surgery.

## 1. Introduction

Adenosarcoma is a rare tumor which consists of benign glandular epithelium and malignant mesenchymal component. This entity was originally described by Clement and Scully [1] in 1974 as Müllerian adenosarcoma. Typically it presents as asolitary large polypoid mass arising from the uterine fundus and fills the endometrial cavity and protrudes from the uterine cervix. Although adenosarcoma is typically low grade tumor, recurrences have been reported in up to 30–40% of patients while 20–25% of women die from their tumors [2]. Here we report a case of adenosarcoma of the uterine corpus.

## 2. Case Report

**Figure 1:**
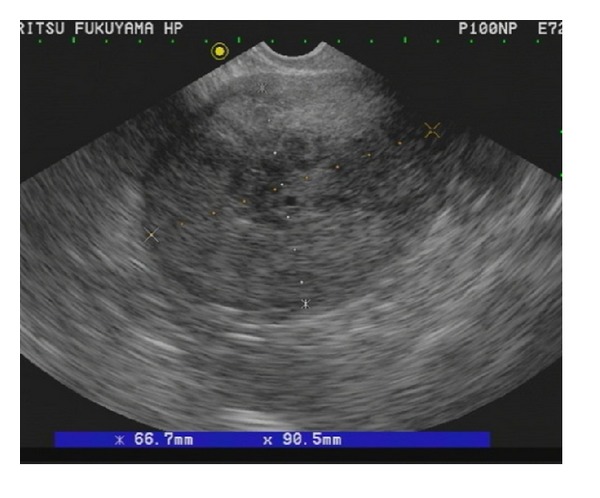
Ultrasound scans revealed a large heterogeneous mass occupying the whole uterine cavity.

A 59-year-old postmenopausal woman, gravida 2, para 2, presented with vaginal bleeding and visited a local clinic. Cytological tests of uterine cervix and endometrium were both negative. She had a uterine tumor pointed out and was referred to our hospital. Vaginal examination revealed enlarged uterus and ultrasound scans revealed a large heterogeneous mass occupying the whole uterine cavity (Figure 1).

**Figure 2:**
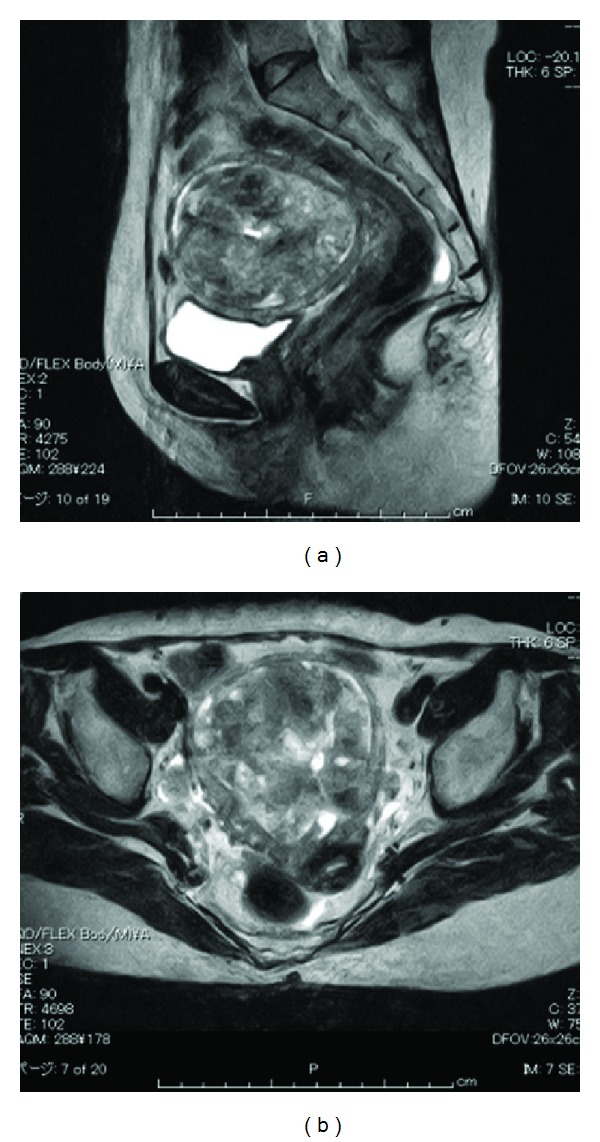
MRI (T_2_-weighted) revealed a heterogeneous solid tumor of 77 × 76 mm.

Cytological test of endometrium was performed again but the result was negative. A fractional endometrial curettage revealed only fibrous tissue with epithelial-like cells. Magnetic resonance imaging (MRI) revealed a heterogeneous solid tumor of 77 × 76 mm (Figure 2).

**Figure 3:**
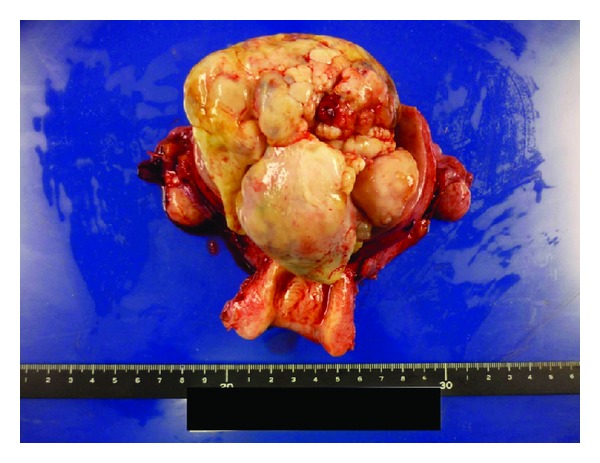
The surgical specimen.

Degenerated myoma, leiomyosarcoma, or endometrial stromal sarcoma was suspected. Serum levels of CA125 were slightly elevated to 41.4 U/mL, whereas CA19-9 and CEA were within normal limits. The patient was admitted and total abdominal hysterectomy with bilateral salpingo-oophorectomy and pelvic lymphadenectomy was carried out (Figure 3).

**Figure 4:**
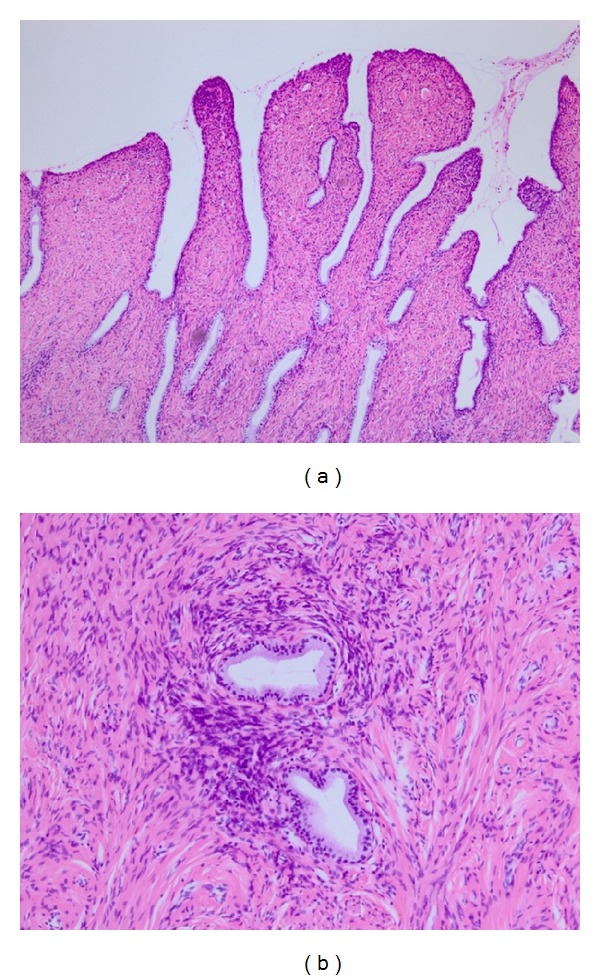
(a) Phyllodes-like architecture on low magnification (H.E. ×100). (b) Periglandular cuffing of tumor cells (H.E. ×400).

At laparotomy, the uterus was fist size and no serosal invasion was observed. Both ovaries were intact. The tumor was arising from the uterine body and occupied the whole uterine cavity. Glandular epithelium with little atypia and proliferation of atypical mesenchymal cells were seen. Mitosis exceeded 2 per 10 high power fields. No myometrial invasion or lymph node metastasis was seen. The lesion was confined to the uterus. Peritoneal cytology revealed no malignant cells. Histopathological final diagnosis was adenosarcoma (Figure 4).

Her postoperative course was uneventful. She was discharged without postoperative treatment and remains alive without disease 6 months after the surgery.

## 3. Discussion

Mixed epithelial-mesenchymal tumors of the uterus include adenofibroma, adenosarcoma, and carcinosarcoma. Adenofibroma has benign glandular epithelial element and benign mesenchymal stroma, whereas carcinosarcoma has both malignant epithelial and mesenchymal stroma. Adenosarcoma is one of the rare diseases consisting of benign glandular epithelial element and malignant mesenchymal component. It may be classified as an intermediate state between the two formerly stated entities. It accounts for 8% of all uterine sarcomas. This entity was originally described by Clement and Scully [1] in 1974 as Müllerian adenosarcoma. Although it usually arises in the endometrium, it can arise in the cervix, the myometrium, fallopian tubes, and ovaries. Typically it presents as a solitary large polypoid mass arising from the uterine fundus and fills the endometrial cavity and protrudes from the uterine cervix [3].

Adenosarcoma is a typically low grade tumor and behave like low grade sarcoma. Adenosarcoma with sarcomatous overgrowth was first used by Clement in 1989 for those tumors that contain more than 25% of sarcomatous component [4]. This is a high grade tumor and runs an aggressive course in contrast with adenosarcoma. Sarcomatous elements are usually homologous, but heterologous elements like rhabdomyosarcoma, cartilage, and skeletal muscle tissue have also been reported [5, 6].

Common symptom is genital bleeding. As for MRI findings, Yoshizakoet al. reported a case of uterine adenosarcoma demonstrated on magnetic resonance (MR) imaging. Imaging revealed a markedly enlarged uterus with thin myometrium occupied by a large polypoid mass. The mass contained solid components with low intensity on T_1_-weighted images and high intensity on T_2_-weighted images compared to the myometrium and areas of small cysts [7]. Takeuchi et al. reported a low grade tumor, which presents as a large polypoid mass occupying the endometrial cavity and protruding into the vaginal cavity. The presence of small hyperintense cysts scattered within the mass on T_2_-weighted imaging, reflecting glandular epithelial components, and relatively low signal intensity on high b value diffusion-weighted imaging, reflecting its low grade nature, may be characteristic findings [8].

Unfavourable prognostic factors are sarcomatous overgrowth, deep myometrial invasion, presence of heterologous elements and extrauterine spread [6]. Tanner et al. reported that, in patients with adenosarcoma, 2-year PFS and OS rates were both 100% compared to 20 for patients with sarcomatous overgrowth. Most patients with adenosarcoma alone survive at least 5 years with surgery alone. In their series, ovarian metastases were not found in patients with uterine adenosarcoma [9]. For premenopausal patients TAH without BSO would be an option.

As for lymphadenectomy, Kaku et al. reported a lymph node metastasis rate of 6.5% and para-aortic lymph node metastasis rate of 0% in 31 patients with adenosarcoma. Two patients with lymph node metastasis had myometrial invasion, heterologous elements, and sarcomatous overgrowth [10]. Tanner et al. reported no lymph node metastases in 11 patients who had lymphadenectomy performed. They suggested that staging lymphadenectomy would not be necessary in patients with disease grossly confined to the uterus and without high risk factors.

There is no optimal adjuvant or systemic treatment strategy but standard sarcoma chemotherapy regimens appear to have efficacy in both adenosarcoma and adenosarcoma with sarcomatous overgrowth [9]. Tanner et al. recommend standard sarcoma regimens such as doxorubicin, ifosfamide, or gemcitabine/docetaxel to patients with measurable adenosarcoma with sarcomatous overgrowth.

Adenosarcoma of the uterus should be a differential diagnosis when a large polypoid mass is occupying the endometrial cavity and protruding into the vaginal cavity. A biopsy specimen often fails to diagnose this entity, and pathological diagnosis should be made on surgical specimen.

## References

[1] Clement PB, Scully RE (1974). Mullerian adenosarcoma of the uterus. A clinicopathologic analysis of ten cases of a distinctive type of mullerian mixed tumor. *Cancer*.

[2] Arend R, Bagaria M, Lewin SN (2010). Long-term outcome and natural history of uterine adenosarcomas. *Gynecologic Oncology*.

[3] Clement PB, Scully RE (1990). Mullerian adenosarcoma of the uterus: a clinicopathologic analysis of 100 cases with a review of the literature. *Human Pathology*.

[4] Clement PB (1989). Mullerian adenosarcomas of the uterus with sarcomatous overgrowth. A clinicopathological analysis of 10 cases. *American Journal of Surgical Pathology*.

[5] Bagga R, Keepanasseril A, Srinivasan R (2010). Adenosarcoma of the uterine cervix with heterologous elements: a case report and review of literature. *Archives of Gynecology and Obstetrics*.

[6] Sinha A, Phukan JP, Sengupta S (2012). Mullerian adenosarcoma of uterus with sarcomatous overgrowth and heterologous component associated with stromal deposit in omentum: a case report and review of the literature. *Case Report in Medicine*.

[7] Yoshizako T, Wada A, Kitagaki H, Ishikawa N, Miyazaki K (2011). MR imaging of uterine adenosarcoma: case report and literature review. *Magnetic Resonance in Medical Sciences*.

[8] Takeuchi M, Matsuzaki K, Yoshida S (2009). Adenosarcoma of the uterus: magnetic resonance imaging characteristics. *Clinical Imaging*.

[9] Tanner EJ, Toussaint T, Leitao Jr MM (2013). Management of uterine adenosarcomas with and without sarcomatous overgrowth. *Gynecologic Oncology*.

[10] Kaku T, Silverberg SG, Major FJ, Miller A, Fetter B, Brady MF (1992). Adenosarcoma of the uterus: a gynecologic oncology group clinicopathologic study of 31 cases. *International Journal of Gynecological Pathology*.

